# CARE-compliant case report: Nemaline myopathy caused by the ACTA1 p.Q139H missense mutation

**DOI:** 10.1097/MD.0000000000045459

**Published:** 2025-11-07

**Authors:** Xinru Pei, Yan Zhai, Fugang Yang, Wei Lu

**Affiliations:** a Interventional Diagnostic and Therapeutic Center, Zhongnan Hospital of Wuhan University, Wuhan, China.

**Keywords:** ACTA1 gene mutation, genetic testing, multidisciplinary supportive care, myopathic changes, nemaline myopathy

## Abstract

**Rationale:**

Nemaline myopathy (NM) is a rare inherited muscle disorder characterized by nemaline rods in muscle fibers and clinically presenting with muscle weakness and hypotonia. This report describes a case of NM caused by a mutation in the ACTA1 (actin alpha 1) gene to enhance the understanding of its diagnosis and clinical course, particularly in the context of pregnancy.

**Patient concerns:**

A 33-year-old female presented with progressive lower limb weakness and muscle atrophy. Symptom onset occurred during pregnancy. Laboratory tests revealed an elevated serum creatine kinase level (1011 U/L).

**Diagnoses:**

Genetic testing identified a heterozygous missense mutation (Q139H, c.417G>C) in exon 3 of the ACTA1 gene, confirming the diagnosis of NM. A muscle biopsy to definitively identify nemaline rods was indicated but was declined by the patient.

**Interventions:**

The diagnostic evaluation included clinical examination, serum creatine kinase testing, electromyography, and genetic analysis. Following diagnosis, the patient was managed with a regimen of supportive care.

**Outcomes:**

Electromyography confirmed myopathic changes. With supportive care, the patient remained clinically stable at follow-up.

**Lessons:**

This case underscores the diagnostic utility of genetic testing for NM when a muscle biopsy is not feasible. It also demonstrates the potential for symptom exacerbation or onset during pregnancy and highlights the importance of multidisciplinary supportive care and genetic counseling in the management of ACTA1-related NM.

## 1. Introduction

Nemaline myopathy (NM) is the most prevalent congenital myopathy,^[1]^ affecting individuals from newborns to adults, with the severity of clinical manifestations closely related to the age of onset.^[2]^ The primary clinical features of myopathy with inclusions included proximal muscle weakness and decreased muscle tone. A pathological hallmark of NM is the presence of linear bodies within muscle fibers.^[3]^ These linear bodies are abnormal electron-dense structures identified in skeletal muscle,^[4]^ which include disordered Z-disks and filament proteins. Such abnormalities may disrupt muscle function, leading to sarcomere dysfunction and subsequent muscle weakness.^[5,6]^ NM is genetically determined and follows both autosomal dominant (AD) and recessive inheritance patterns.^[7]^ Mutations in actin alpha 1 (ACTA1) and nebulin are the most common etiological factors associated with NM.^[8]^

In this report, we describe a case of NM resulting from a missense mutation in the ACTA1 gene. Currently, the patient exhibits muscle swelling and hypotonia in both lower limbs, accompanied by decreased skin temperature in the bilateral gluteal region. Neurological and systemic examination results were unremarkable.

## 2. Case presentation

This study was approved by the Human Ethical Committee of Zhongnan Hospital of Wuhan University, Wuhan, China. Written informed consent was obtained from the patient for publication of this case report. Written informed consent was obtained from the patient for publication of this case report and any accompanying images.

We report the case of a 33-year-old female who presented with progressive lower limb weakness and motor impairment. Her symptoms began 5 years ago during pregnancy with a sensation of acid swelling in both lower limbs. At that time, laboratory tests revealed elevated creatine kinase (CK) and creatine kinase-myocardial band (CK-MB) levels, but no further investigation was pursued. Two years ago, the lower limb swelling worsened and was accompanied by progressive muscle weakness, leading to difficulty in climbing stairs, for which she required external assistance.

On physical examination, the patient was alert, well-developed, and had a normal gait. Upper limb strength was normal. However, muscle strength in both lower limbs was graded 4/5, with slightly reduced muscle tone. Knee reflexes were diminished bilaterally. Sensation, including superficial, deep, pain, temperature, and proprioception, was intact. Challenging maneuvers such as climbing stairs, squatting, and rising from a seated position were difficult. Mild muscle atrophy was observed in the thigh and calf muscles, but there was no evidence of scoliosis or pathological reflexes.

**Figure 1.**
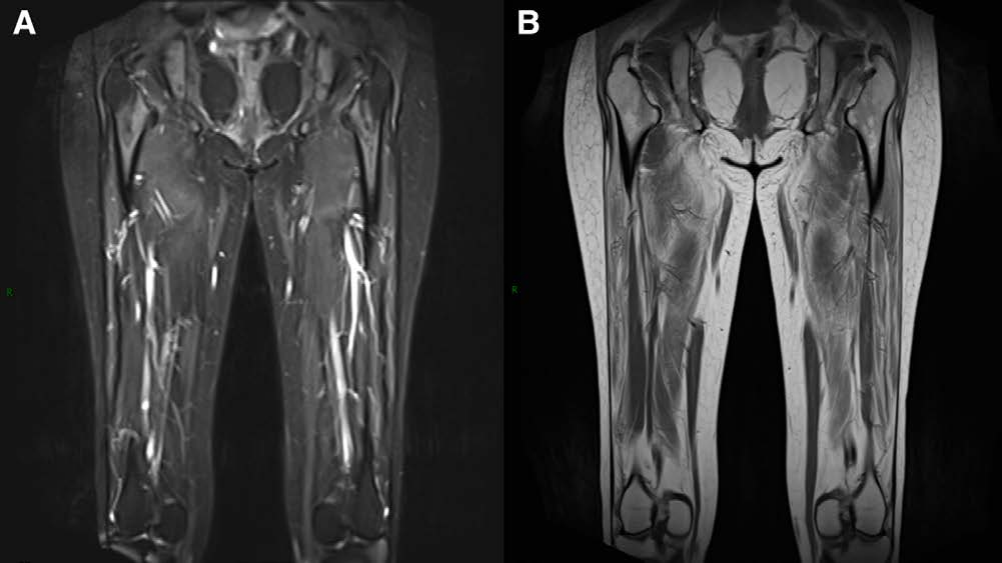
MRI results of the patient’s bilateral thighs. The shape of the bilateral femurs was normal on MRI, and an uneven T2 fat-suppressed high signal consistent with edema was seen in the upper medullary cavity with unclear margins (A). Bilateral long T2WI signal intensity suggestive of myopathic changes was observed in the lower segment of the vastus medialis muscles (B). MRI = magnetic resonance imaging.

Diagnostic evaluation included serological, electrophysiological, imaging, and genetic studies. Laboratory tests revealed elevated serum CK (1011 U/L) and CK-MB (12.6 ng/mL). Nerve conduction studies were normal in both upper and lower limbs (Tables S1–S3, Supplemental Digital Content, https://links.lww.com/MD/Q515). In contrast, electromyography showed abnormal spontaneous activity (fibrillations and positive sharp waves) and myopathic motor unit potentials (short duration, low amplitude, increased polyphasia; Table S4, Supplemental Digital Content, https://links.lww.com/MD/Q515). Magnetic resonance imaging of the thighs revealed edema and fatty infiltration suggestive of myopathic changes in the bilateral femoral muscles (Fig. 1). Lower limb vascular ultrasonography was unremarkable. Electrocardiography and thyroid function tests were normal.

Genetic testing identified a heterozygous missense mutation in exon 3 of the ACTA1 gene (c.417G>C, p.Q139H; Table 1), a known pathogenic variant associated with NM.^[9]^ A muscle biopsy was offered to the patient to provide histopathological confirmation of nemaline rods; however, after considering the invasive nature of the procedure, the financial burden, and the established genetic diagnosis, the patient declined.

**Table 1:** Genetic test results of the patient.

Gene and transcript IDs	Chromosome location	Gene subregion	Variation information	Name of disease	Genetic pattern	Zygotic type	Variation evaluation
ACTA1 (NM_001100.4)	chr1:229 568340	exon3	c.417G>C (p.Q139H)	Congenital myopathy type 2A (OMIM:161800) Congenital myopathy type 2B (OMIM:620265) Congenital myopathy type 2C (OMIM:620278)	AD AR AD	Heterozygosis	Pathogenicity
MTM1 (NM_000252.2)	chrX:149 761121	exon2	c.45G>T (p.E15D)	X-linked central nuclear myopathy (OMIM:310400)	XLR	Heterozygosis	Ambiguity of meaning
TNNT1 (NM_003283.6)	chr19:55 648531	exon11	c.551G>A (p.R184H)	Nemaline myopathy type 5A (OMIM:605355) Nemaline myopathy type 5B (OMIM:620386) Nemaline myopathy type 5C (OMIM:620389)	AR AR AD	Heterozygosis	Ambiguity of meaning

Genetic testing identified a heterozygous missense mutation in exon 3 of the ACTA1 gene (c.417G>C, p.Q139H).

AD = autosomal dominant, AR = autosomal recessive, XLR = X-linked recessive.

**Figure 2.**
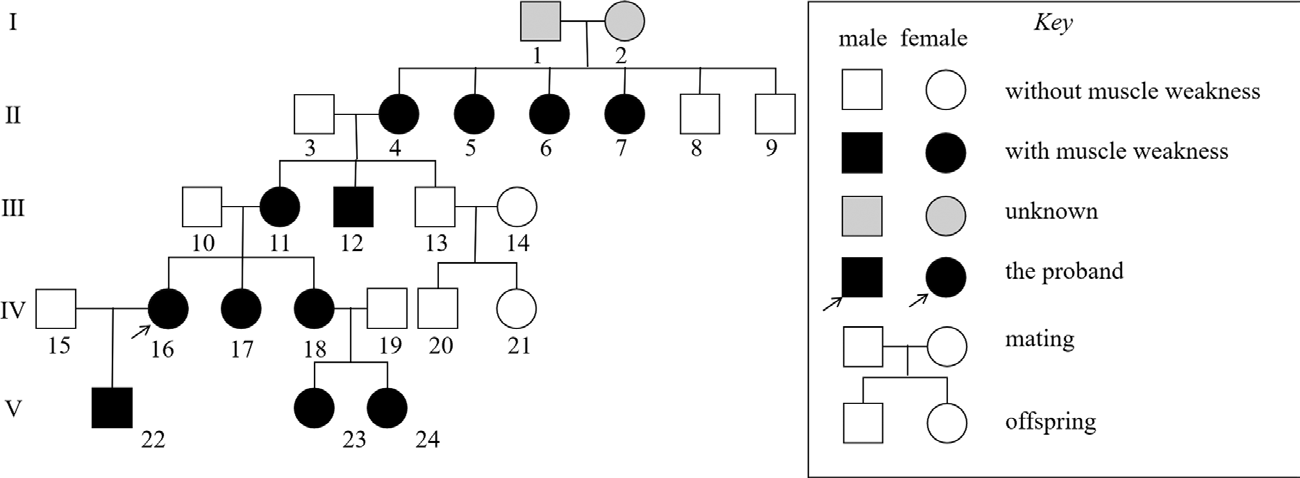
Pedigree map of the patient’s family members. The patient represents the first confirmed case in a family with multiple members showing myasthenic manifestations.

The patient’s family history was significant for similar myopathic manifestations in multiple relatives (Fig. 2), suggesting an AD inheritance pattern, though this could not be genetically confirmed in other family members. In the absence of acute complications or disease-modifying therapies, the patient received supportive care and was discharged. Telephone follow-up confirmed stable disease status post-discharge. The patient reported adapting to functional limitations but expressed concerns about disease progression and genetic risks for her offspring.

A timeline summarizing the key clinical events and diagnostic findings is provided in Table 2.

**Table 2:** Diagnostic and management milestones.

Timeline	Clinical event	Diagnostic
2018	Pregnancy-onset limb heaviness	CK/CK-MB testing (elevated)
2021	Progressive weakness	Initial clinical evaluation
2023	Lower limb weakness (4/5) MRI bilateral thigh inflammation Genetic testing results	Serum CK (1011 U/L), EMG (myopathic changes) Muscle MRI with T2 fat-suppressed sequences ACTA1 p.Q139H mutation identified

The case began with pregnancy-onset limb heaviness (2018) and elevated creatine kinase (CK) levels, followed by progressive weakness (2021). By 2023, the patient exhibited lower limb weakness (Medical Research Council grade 4/5), muscle inflammation on MRI, and confirmation of an ACTA1 p.Q139H mutation through genetic testing.

ACTA1 = actin alpha 1, CK = creatine kinase, CK-MB = creatine kinase-myocardial band, EMG = electromyography, MRI = magnetic resonance imaging.

## 3. Discussion

NM is the most prevalent congenital myopathy and is characterized by the presence of eosinophilic rod-shaped inclusions or linear bodies within the muscle fibers.^[3]^ To date, pathogenic mutations have been identified in at least 14 genes.^[10]^ The majority of NM patients possess mutations in the nebulin gene (approximately 50%) or the ACTA1 gene (20–30%), with ACTA1 mutations being most prevalent among those with severe congenital onset.^[11–13]^ ACTA1 encodes skeletal muscle α-actin (42 kDa), which interacts with myosin during muscle contractions. Mutations in ACTA1 disrupt the normal structure and function of α-actin, resulting in muscle weakness, hypotonia, and a spectrum of muscle conditions collectively termed “actinopathies.”^[14,15]^

NM is a group of diseases with genetic and clinical heterogeneity, and is usually characterized by nonprogressive or slowly progressive generalized muscle weakness. The clinical presentation of NM can vary widely and individuals with the same mutation in the same gene may exhibit different severities.^[16]^ Understanding the clinical characteristics of patients with NM is crucial for recognizing the limitations of diagnosing this rare disease and providing appropriate treatment.^[17]^

According to the 1999 European Neuromuscular Centre classification,^[1]^ the adult-onset of our patient’s symptoms, initially manifesting during pregnancy, supports a diagnosis of adult-onset NM. This diagnosis was genetically confirmed by the identification of a heterozygous p.Q139H missense mutation in the ACTA1 gene, a known pathogenic variant,^[9,18]^ supported by elevated CK levels and myopathic features on electromyography and magnetic resonance imaging. Notably, while the p.Q139H variant has been predominantly linked to severe congenital NM with intranuclear rods,^[9]^ Our patient’s markedly milder, adult-onset phenotype, which manifested following the physiological stresses of pregnancy (e.g., fluid retention, weight gain, and hormonal changes), demonstrates how such stressors can interact with a genetic predisposition to unmask subclinical disease, underscoring the considerable phenotypic heterogeneity of ACTA1-related myopathies. Although cardiac involvement can be observed in a small number of NM patients with ACTA1 mutations,^[19]^ the elevated CK-MB in our patient is most plausibly attributed to the extensive skeletal muscle damage, given the absence of cardiac symptoms or electrocardiographic abnormalities. The lack of baseline cardiac imaging (e.g., echocardiogram) precludes a definitive assessment of cardiac status; thus, future cardiac surveillance is warranted if relevant symptoms emerge. Additionally, several family members of the patient had similar histories; however, the patient refused genetic testing for the rest of the family. Therefore, we could not determine the inheritance pattern of this disease in the family, though the pedigree is suggestive of AD transmission.

This case contributes to the literature by describing the course of NM associated with the ACTA1 p.Q139H variant, particularly highlighting the potential for adult-onset and symptomatic exacerbation during pregnancy. Muscle biopsy, the historical gold standard revealing nemaline rods, was not performed as the patient declined. While this limits histopathological confirmation, the combination of clinical presentation, electrophysiological findings, and identification of a known pathogenic ACTA1 variant provides strong support for the diagnosis. It underscores the utility of genetic testing in achieving a diagnosis when biopsy is not available. The challenges encountered, including the patient’s refusal of biopsy and family testing, reflect real-world limitations in managing rare diseases and highlight areas for improving patient education and counseling. The main limitation of this study is the lack of histopathological confirmation and functional validation of the genetic variant. Future studies should focus on functional characterization of such variants and exploring potential genotype-phenotype correlations. Long-term multidisciplinary follow-up is essential to manage the progressive nature of this condition and address reproductive genetic concerns. The long-term management of NM necessitates a proactive, stage-specific approach. In the early ambulatory phase, interventions focus on fall prevention and physical therapy to preserve function. With progression, care must shift to preventing complications of impaired mobility, such as pressure sores, and addressing potential dysphagia. In advanced stages, the paramount challenge is the prevention of respiratory failure through vigilant monitoring. This case underscores that effective management anticipates these specific challenges at each disease stage to optimize patient quality of life.

## 4. Conclusion

The diagnosis of NM is often made through genetic testing, combined with supportive muscle biopsy findings of characteristic pathological alterations of “nemaline bodies” in the muscle. Currently, there is no cure for patients with NM, and treatment is mainly supportive and symptomatic. Physical therapy can help alleviate symptoms, strengthen muscles, and improve functional abilities. Regular monitoring of respiratory and cardiac function is important to prevent fatal accidents. Comprehensive management and support are essential for quality of life and long-term survival of patients with NM. Through this case report, we hope to enhance clinicians’ understanding of the clinical presentation and genetic basis of NM, particularly the diagnostic challenges and the importance of genetic counseling in families with inherited myopathies. Further research into the mechanisms underlying ACTA1 mutations and potential therapeutic strategies is warranted.

## Acknowledgments

We thank the patient for this report and her family members.

## Author contributions

**Conceptualization:** Xinru Pei, Wei Lu.

**Data curation:** Xinru Pei, Yan Zhai, Fugang Yang.

**Investigation:** Yan Zhai, Fugang Yang, Wei Lu.

**Methodology:** Fugang Yang.

**Resources:** Wei Lu.

**Supervision:** Wei Lu.

**Visualization:** Xinru Pei, Yan Zhai.

**Writing** – **original draft:** Xinru Pei.

**Writing** – **review & editing:** Xinru Pei.

## Supplementary Material


