# Supplementary material for: CARE-compliant case report: Nemaline myopathy caused by the ACTA1 p.Q139H missense mutation
Source: Medicine (Baltimore). 2025 Nov 7;104(45):e45459. doi: 10.1097/MD.0000000000045459 (PMC12599729; doi:10.1097/MD.0000000000045459)
Supplement: Supplementary file 1 [file medi-104-e45459-s001.docx]

Table S1 | Sensory Neuron Conduction studies of the patient.

| **Sensory Neuron Conduction Speed(SNCS)** | | | | | |
| --- | --- | --- | --- | --- | --- |
| Nerve (Left) | | Peak Lat | Amp | CV | Dist |
|  |  | ms | uV | m/s | mm |
| Ulnar nerve | Wrist-FingerⅤ | 1.92 | 44.1 | 52.1 | 100 |
| Median nerve | Wrist-FingerⅡ | 2.07 | 50.8 | 58.0 | 120 |
| Superficial peroneal nerve | Lower leg-Anklk | 1.54 | 30.8 | 58.4 | 90.0 |
| Peroneal intestinal nerve | Calf-Lateral malleolus | 1.50 | 25.6 | 56.7 | 85.0 |

The sensory conduction of the median and ulnar nerves in the left upper limb, as well as the superficial peroneal and sural nerves in the left lower limb, showed no abnormalities.(Peak Lat=Peak Latency, Amp=Amplitude, CV=Conduction Velocity, Dist=Distance.)

Table S2 | F-Wave studies of the patient.

| **F-Wave** | | | | | | | |
| --- | --- | --- | --- | --- | --- | --- | --- |
| Nerve (Left) | | M-Lat | F-Lat(mean) | Amp | F% | Dist | CV |
|  |  | ms | ms | uv | % | mm | m/s |
| Median nerve | Wrist-APB | 3.3 | 24.6 | 355 | 100 |  | -- |
| Tibial nerve | Ankle-AH | 1.54 | 44.2 | 348 | 100 |  | -- |

The F waves of the median nerve in the left upper limb and the tibial nerve in the left lower limb were normal.(M-Lat=Motor Latency, F-Lat=F-wave Latency, Amp=Amplitude, F%=F-wave Persistence/Persistence Ratio, Dist=Distance, CV=Conduction Velocity, Wrist-APB=Wrist to Abductor Pollicis Brevis, Ankle-AH=Ankle to Abductor Hallucis.)

Table S3 | Motor Neuron Conduction studies of the patient.

| **Motor Neuron Conduction Speed(MNCS)** | | | | | |
| --- | --- | --- | --- | --- | --- |
| Nerve (Left) | | Lat | Amp | CV | Dist |
|  |  | ms | mV | m/s | mm |
| Ulnar nerve | Wrist-ADM | 2.33 | 10.4 |  | 45.0 |
|  | Infra-elbow-Wrist | 5.25 | 10.1 | 59.9 | 175 |
|  | Supra-elbow-Infra-elbow | 7.22 | 9.0 | 50.8 | 100 |
| Median nerve | Wrist-APB | 3.33 | 6.9 |  | 45.0 |
|  | Elbow-Wrist | 6.83 | 6.2 | 60.0 | 210 |
| Tibial nerve | Ankle-AH | 2.83 | 11.9 |  | 65.0 |
| Common peroneal nerve | Ankle-EDB | 3.46 | 6.4 |  | 60.0 |
|  | BI Fib.head-Ankle | 9.79 | 6.1 | 53.7 | 340 |
|  | Ab Fib.head-BI Fib.head | 11.2 | 5.9 | 42.6 | 60.0 |

The motor conduction of the median and ulnar nerves in the left upper limb, as well as the tibial and common peroneal nerves in the left lower limb, showed no abnormalities.(Lat=Latency, Amp=Amplitude, CV=Conduction Velocity, Dist=Distance; Wrist-ADM=Wrist to Abductor Digiti Minimi, Wrist-APB=Wrist to Abductor Pollicis Brevis, Ankle-AH=Ankle to Abductor Hallucis, Ankle-EDB=Ankle to Extensor Digitorum Brevis, BI Fib.head-Ankle=Biceps Femoris (long head) to Fibular head-Ankle, Ab Fib.head-BI Fib.head=Above Fibular head-Biceps Femoris (long head) to Fibular head.)

Table S4 | Electromyography Results of the patient.

| **Electromyography Results** | | | | | | | |
| --- | --- | --- | --- | --- | --- | --- | --- |
| Muscle(Left) | Spontaneous Activity | | Voluntary Activity | | | | |
|  | Fib | PSW | Amp | Dur | Poly | Stabil | IP |
| First dorsal interosseous muscle | 0/10 | 0/10 | Normal | Normal | + | Normal | Normal |
| Biceps | 0/10 | 0/10 | Normal | Normal | + | Normal | Normal |
| Deltoid | 0/10 | 1/10 | Normal | - | Normal | Normal | + |
| Tibialis anterior | 0/10 | 1/10 | - | - | + | Normal | + |
| Rectus femoris | 2/10 | 3/10 | Normal | - | + | Normal | + |

The EMG of the left anterior tibialis, medial head of the quadriceps femoris, and deltoid muscle showed abnormal spontaneous activity, and the MUP (motor unit potential) analysis indicated myogenic damage. The EMG of the left first dorsal interosseous muscle and biceps brachii showed no abnormal spontaneous activity, and the MUP analysis indicated myogenic damage.(Fib=Fibrillations, PSW=Positive Sharp Waves, Amp=Amplitude, Dur=Duration, Poly=Polyphasic Potentials, Stabil=Stability, IP=Insertional Activity.)
